# Clinical efficacy of adjunctive methods for the non-surgical treatment of peri-implantitis: a systematic review and meta-analysis

**DOI:** 10.1186/s12903-023-03058-z

**Published:** 2023-06-09

**Authors:** Luigi Barbato, Raffaele Cavalcanti, Cosimo Rupe, Daniele Scartabelli, Lapo Serni, Leandro Chambrone, Francesco Cairo

**Affiliations:** 1 Department of Clinical and Experimental Medicine, Research Unit in Periodontology and Periodontal Medicine, University of Florence (Italy), Via Casentino, 29 Florence, Italy; 2 Department of General Surgery and Surgical-Medical Specialties, University of Catania (Italy), Catania, Italy; 3 Evidence-Based Hub, Egas Moniz Center for Interdisciplinary Research, Egas Moniz School of Health & Science, Almada, Portugal; 4 Unit of Basic Oral Investigation (UIBO), Universidad El Bosque, Bogotá, Colombia; 5 Department of Periodontics, School of Dental Medicine, The University of Pennsylvania, Philadelphia, PA USA

**Keywords:** Peri-implantitis, Non-surgical treatment, Peri-implant debridement, Adjunctive methods, Systemic antibiotics, Submarginal instrumentation

## Abstract

**Background:**

The aim of this systematic review (SR) was to evaluate the clinical efficacy of different adjunctive methods/therapies to the non-surgical treatment (NST) of peri-implantitis.

**Materials and methods:**

The protocol of the review was registered in PROSPERO database (CRD42022339709) and was designed according to PRISMA statement. Electronic and hand searches were performed to identify randomized clinical trials (RCTs) comparing non-surgical treatment of peri-implantitis alone versus NST plus any adjunctive method/treatment. The primary outcome was probing pocket depth (PPD) reduction.

**Results:**

Sixteen RCTs were included. Only 2 out of 1189 implants were lost and follow-up ranged from 3 to 12 months. PPD reduction across the studies varied from 0.17 to 3.1 mm, while defect resolution from 5.3% to 57.1%. Systemic antimicrobials were associated to higher PPD reduction (1.56 mm; [95% CI 0.24 to 2.89]; *p* = 0.02) with high heterogeneity, and treatment success (OR = 3.23; [95% CI 1.17 to 8.94]; *p* = 0.02), compared to NST alone. No differences were found with adjunctive local antimicrobials and lasers for PPD and bleeding on probing (BoP) reduction.

**Conclusions:**

Non-surgical treatment with or without adjunctive methods may reduce PPD and BoP even if complete resolution of the pocket is unpredictable. Among possible adjunctive methods, only systemic antibiotics seems to provide further benefits, but their usage should be considered with caution.

**Supplementary Information:**

The online version contains supplementary material available at 10.1186/s12903-023-03058-z.

## Introduction

Peri-implantitis is a plaque-associated disease occurring around dental implants, characterized by inflammation of the peri-implant mucosa and progressive bone loss [1, 2]. Peri‐implantitis exhibits clinical signs of inflammation (i.e., bleeding on probing [BoP] and/or suppuration), increased probing pocket depth [PPD] and/or recession of the mucosal margin in addition to progressive loss of supporting bone [3].

It is suggested that peri-implantitis progresses in a non-linear, accelerating pattern and that the onset occurs, usually, within 3 years of function [4, 5]. History of periodontitis, poor plaque control, and irregular supportive peri-implant care represent the main risk factors for peri-implantitis [6, 7]. The role of other risk factors remains unclear [8].

It is difficult to define the global prevalence of peri-implantitis, mostly due to the wide range of case definitions used across studies [9]. The available evidence shows that prevalence of periimplantitis ranges between 15 and 56% [4, 8, 10]. Recent data showed an implant loss due to peri-implantitis up to 13.6% at the patient level and up to 8.3% at the implant level [10, 11].

Treatment of peri-implantitis remains challenging for the clinicians. A recent study with 5 years follow-up reported 44% recurrence/progression of peri-implantitis, and 27% implant loss also after the treatment. Residual PPD ≥ 6 mm was the main risk factor for recurrence and progression of peri-implantitis, highlighting the need for a proper intervention in case of peri-implantitis [12].

Both non-surgical and surgical interventions were proposed to treat peri-implantitis [13]. However, the treatment should start from the infection control procedures [14]. The decontamination of the implant surface is more challenging and unpredictable compared to the treatment on natural teeth [15]. On this way, the adjunctive use of lasers [16], systemic or local antibiotics [17] and antimicrobial photodynamic therapy (aPDT) [18], have been proposed and investigated in both pre-clinical and clinical studies in order to increase implant surface decontamination.

Therefore, the aim of this systematic review (SR) was to evaluate the clinical efficacy of different adjunctive methods/therapies to the non-surgical treatment (NST) of peri-implantitis.

## Materials and methods

This SR was reported in accordance with the PRISMA statement [19]. The review protocol was registered in PROSPERO database (CRD42022339709).

### Focus question

PICO method and the guidelines of the Center for Evidence-Based Medicine (University of Oxford, Oxford, UK) [20] were used, and the focused question was: “*In patients with peri-implantitis, what is the additional benefit of adding adjunctive methods to non-surgical therapy compared with to non-surgical therapy alone?”.*

#### Population

Adult patients, affected by peri-implantitis, not affected by systemic diseases (i.e., diabetes, autoimmune diseases).

#### Intervention

Non-surgical implant surface debridement.

#### Comparison

Any adjunctive treatment to the non-surgical implant surface debridement.

#### Outcomes

The primary outcome was PPD reduction. Secondary outcomes were: relative clinical attachment level (RAL) gain, marginal bone level (MBL) changes, BoP reduction, composite outcome for treatment success/resolution, Implant Survival.

#### Eligibility criteria

- Only randomized clinical trials (RCTs) treating at least 5 patients per group and reporting PPD reduction in millimeters at final follow-up visit were considered.
- Each patient had at least one dental implant suffering from peri-implantitis, treated by means of non-surgical debridement with or without adjunctive methods.
- A diagnosis of peri-implantitis in accordance with the 2017 classification [3]. Studies published or designed before 2017 were included only if the diagnosis of peri-implantitis was based on the same criteria of the 2017 classification.
- Minimum follow-up was 3 months.
- Only English language articles were considered.

### Information sources and search strategy

Three on-line databases (PUBMED, EMBASE, and COCHRANE) were used. The last search was on October 20, 2022. (For detail information on the search strategy see Additional file 1).

Following journals were hand searched from July 2012 to October 2022, *Clinical Implant Dentistry and Related Research, Journal of Clinical Periodontology, Journal of Periodontology, Journal of Periodontal Research, Journal of Dental Research, Clinical Oral Implant Research*. The references list of the included studies and other relevant SRs on the topic were also examined for additional papers.

### Selection process

Studies' eligibility was independently assessed by two reviewers (C.R. and D.S.). Records were screened by title and abstract after duplicates removal, then the eligible papers were evaluated in full text. In case of disagreement, a third reviewer (L.B.) was involved for the final decision. The level of agreement between the two examiners was evaluated using Cohen's kappa coefficient (k).

### Data collection process and data items

A customized table was used by two reviewers (C.R. and D.S.) to retrieve information and data from the included studies. For each study, the following information were considered: authors, year of publication, country, definition of peri-implantitis, follow-up, n. of patients, n. of implants, type of treatment, diagnostic criteria for peri-implantitis and outcomes of interest (PPD Reduction, RAL Gain, MBL Changes, BoP changes, treatment success/resolution, Implant Survival). In case of missing or unclear data authors were contacted to clarify any doubt.

### Risk of bias assessment

The risk of bias of the included studies was evaluated using the Cochrane Collaboration’s Tool RoB 2.0 [21]. Briefly, 5 domains (Risk of bias arising from the randomization process, Risk of bias due to deviations from the intended interventions, Risk of bias due to missing outcome data, Risk of bias in measurement of the outcome, Risk of bias in selection of the reported result) were evaluated [21]. Each included study was rated as:

A. Low risk of bias (plausible bias unlikely to seriously alter the results) if all criteria were met.
B. Some concerns risk of bias (plausible bias that raises some doubt about the results) if one or more criteria were partly met.
C. High risk of bias (plausible bias that seriously weakens confidence in the results) if one or more criteria were not met.

### Effect measures and synthesis methods

Studies were initially grouped by characteristics and according to the type of adjunctive treatment. A meta-analysis was performed in case of at least two studies of similar design. Differences between groups were expressed as weighted mean differences (WMD) and 95% confidence interval (CI) for continuous outcomes and odds ratio (OR) and 95% CI for dichotomous outcomes. Standard deviation from the mean differences were calculated when not available, according to Cochrane Handbook for Systematic Reviews of Interventions [22]. Random-effect model was used, and the variables were registered at implant level. The arms were weighted according to the treated sample size [23]. For studies with multiple intervention groups, groups were combined to create a single pair-wise comparison, as recommended by the Cochrane Handbook for Systematic Reviews of Interventions [22].

The heterogeneity was assessed by means of the I^2^ statistics (0%–40% low heterogeneity, 30%–60% moderate heterogeneity, 50%–90% substantial heterogeneity and 75%–100% considerable heterogeneity) [24].

The statistical analyses were carried out using the RevMan software version 5.3 (Copenhagen: The Nordic Cochrane Centre, The Cochrane Collaboration, 2014) and the STATA software package (version 15.0).

## Results

### Study selection

**Fig. 1:**
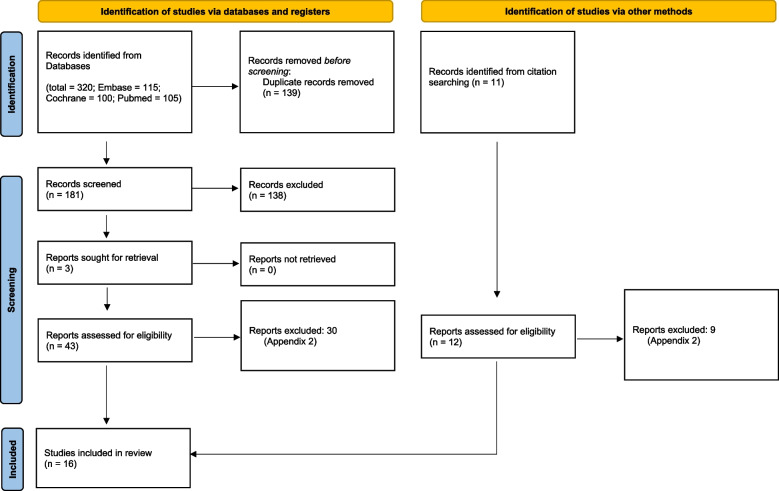
Search results, according to Page MJ, McKenzie JE, Bossuyt PM, Boutron I, Hoffmann TC, Mulrow CD, et al. The PRISMA 2020 statement: an updated guideline for reporting systematic reviews. BMJ 2021;372:n71

The search strategy identified 320 potentially eligible articles. After screening of titles and/or abstracts, 43 articles were selected for full text evaluation and 12 additional papers were retrieved from hand search. Thus, 55 articles were subjected to the eligibility process. Finally, 16 articles were included in the review (Fig. 1) [17, 25–39]. For details on excluded full-text articles see Additional file 2. Cohen's kappa value for the global inter-reviewer agreement was 0.83.

### Study characteristics and results of individual studies

RCTs included in this SR were published between 2012 and 2022. For additional information about country, setting and funding, see Additional file 3.

Follow-up was 3 months in four studies [33, 35, 38, 39], 6 months in six studies [26, 27, 29, 31, 32, 36], 12 months in two studies [28, 30] and data at 3 and 6 months were reported in 3 studies [25, 34, 37] while one study reported data at 6 and 12 months [17].

Initial supragingival professional hygiene was performed in every study before the treatment. All the papers specified that the patients were given detailed instructions for self-plaque control measures before starting the treatment.

Adjunctive therapies used were local antibacterial agents, systemic antibiotics, lasers, aPDT, probiotics and a desiccant agent. Three studies evaluated the adjunctive effect of systemic antibiotics, but using different drugs or dosage: metronidazole 500 mg 3/die for seven days [17]; metronidazole 400 mg and amoxicillin 500 mg, 3/die for 14 days [28]; metronidazole 250 mg and amoxicillin 375 mg three times a day for 7 days [33].

Local antibacterial agents were investigated in four studies. In particular, two studies tested multiple applications of chlorhexidine chips [26, 27] one tested the application of chloramine [38], and another study evaluated the application of minocycline with or without metronidazole [39]. Moreover, different lasers were tested in five studies [29, 37, 32, 30, 34] and two studies used the aPDT as adjunctive method to the debridement alone [35, 36]. Topical application of a dual-strain *Lactobacillus Reuteri* probiotic was tested in one RCT [25]. Finally, Merli et al. performed a three arms RCT evaluating both an air-polishing device and a desiccant material [31].

Only two out of 1189 implants with peri-implantitis treated in the studies were lost during the follow-up. PPD reduction was reported in all group testing debridement alone and varied from 0.2 mm to 1.8 mm. For adjunctive therapies, the use of diode laser was associated to the worst PPD reduction of 0.17 mm while the greatest one was 3.1 mm for systemic antibiotics. Ten studies reported data on BoP changes: BoP was reduced from 10 to 50%. Radiographic bone level changes were reported in three studies showing improvement for both control and test group.

Only 6 studies used a composite outcome for treatment success and the definition was homogeneous (absence of PPD ≥ 5 mm [27], absence of 5 mm PPD associated with BoP and no progressive bone loss [17, 31, 33, 34, 39]). None of the included studies reported a 100% resolution rate, with the success ranging between 5.3% and 57.1%.

The main outcomes are summarized in Table 1. Secondary outcomes are reported in Additional file 4.

**Table 1:** Results of the individual studies

**Authors**	**N° of patients (n° of implants)**		**F.up (months)**	**Treatment**		**Implant failure**	**Treatment success n (%)**		**PPD reduction (mm, SD)**		**BoP reduction (%, SD)**	
	Control	Test		Control	Test		Control	Test	Control	Test	Control	Test
Al – Askar 2022 [35]	16 (16)	17 (17)	3	MD	MD + PBMT	0	-	-	1.7 (0.19)	2.2 (0.57)	-	-
		16 (16)	3	MD	MD + PDT		-	-	-	1.8 (0.06)	-	-
Alpaslan 2022 [32]	17 (17)	33 (33)	6	MD	MD + Laser (Er,Cr:YSGG/dLaser)	0	-	-	0.53 (0.44)	1.01 (0.61)	11.31 (21.58)	37.2 (28.01)
Alqahtani 2019 ^a^ [36]	49 (49)	49 (49)	6	MD	MD + aPDT	0	-	-	1.09 (0.12)	1.09 (0.09)	8.7 (13.6)	12 (19.9)
Alqahtani 2020 ^b^ [37]	34 (34)	33 (33)	6	MD	MD + PBMT	0	-	-	-	-	-	-
Arisan 2015 [29]	5 (24)	5 (24)	6	MD	MD + dLaser	0	-	-	0.21 (0.24)	0.17 (0.41)	-	-
Blanco 2022 ^a^ [17]	16 (28)	16 (34)	12	MD	MD + SA (MTZ)	0	7 (23.5)	19 (57.1)	1.02 (0.48)	2.53 (0.58)	-	-
Laleman 2020 [25]	8 (8)	6 (6)	6	MD	MD + Probiotic	0	-	-	1.27 (1.00)	1.02 (0.69)	33 (27)	27 (23)
Machtei 2012 [27]	30 (37)	26 (36)	6	MD	MD + CHXc	0	6 (15)	10 (28.5)	1.59 (0.23)	2.19 (0.24)	45.5 (8.8)	57.5 (7.92)
Machtei 2021 [26]	144 (189)	146 (197)	6	MD	MD + CHXc	0	-	-	1.54 (1.13)	1.76 (1.13)	55.21	50.31
Merli 2020 [31]	16 (15)	15 (15)	6	MD	MD + Dm	0	6 (37)	4 (25)	0.2 (0.7)	0.5 (0.9)	13.8	20
		13 (15)	6		MD + Gp	2	-	2 (14)	-	0.1 (0.8)	-	25
		14 (15)	6		MD + Gp + Dm	0	-	6 (43)	-	0.8 (0.8)	-	29.6
Park 2021 [39]	37 (37)	38 (38)	3	MD	MD + MTZ + MIN	0	1 (2.7)	12 (31.6)	1.28 (1.15)	1.95 (1.28)	5.5 (6.8)	8.5 (5.3)
		39 (39)	3		MD + MIN	0		8 (20.5)		1.88 (1.5)		8.3 (5.6)
Polymeri 2022 [33]	19 (19)	18 (18)	3	MD	MD + SA (AMX + MTZ)	0	1 (5.3)	1 (5.5)	1.47 (1.95)	2.28 (1.49)	11	16
Roccuzzo 2022 [34]	13 (13)	12 (12)	6	MD	MD + dLaser	0	6 (46.2)	5 (41.7)	1.47 (0.68)	1.28 (0.70)	15.4 (31.5)	15.3 (30.5)
Roos-Jansåker 2017 [38]	16^c^ (16)	16^c^ (16)	3	MD	MD + Chloramine	0	-	-	1.6^d^	1.75^d^	11 (5.5)	9.9 (6.3)
Shibli 2019 [28]	20 (20)	20 (20)	12	MD	MD + SA (AMX + MTZ)	0	-	-	1.8 (0.2)	3.1 (1.2)	-	-
Strauss 2021 [30]	10 (15)	10 (19)	12	MD	MD + Laser (Nd:YAG)	0	-	-	1.36 (2.01)	1.89 (1.33)	-	-

*F.up* follow-up, *PPD* pocket probing depth, *BoP* Bleeding on Probing, *RAL* Relative Attachment Level, *MBL* Marginal Bone Loss, *MD* Mechanical Debridement, *CHXc* Chlorhexidine chips, *SA* Systemic Antibiotics, *MTZ* Metronidazole; dLaser: diode Laser, *Nd:YAG* neodymium-doped yttrium aluminum garnet, *Dm* Dessiccant Material, *Gp* Glycine Powder, *Er,Cr:YSGG* Erbium, chromium-doped yttrium, scandium, gallium garnet, *AMX* Amoxicilline, *PBMT* Photobiomodulation Therapy, *PDT* Photodynamic Therapy, *aPDT* antimicrobial photodynamic therapy, *MIN* Minocycline

^a^For these studies, minimum and maximum values of the different measurements were used to calculate the SD

^b^For this study, baseline and final measurements were reported on graphs

^c^Split-mouth study. Sixteen patients were the total sample

^d^Median values

### Definitions of Peri-implantitis

The included RCTs defined peri-implantitis according to the following parameters: PPD, BoP and evidence of radiographic peri-implant bone loss. Although all studies provided a definition of peri-implantitis consistent with the 2017 classification, slight differences were retrieved between the different papers. In order to provide reliable information to the reader, the definitions used by each study for the diagnosis of peri-implantitis were reported in Table 2.

**Table 2:** Definition of Peri-implantitis in the included studies

*Authors*	*PPD*	*BoP*	*MBL*
Al-Askar 2022 [35]	-	Yes	≥ 3 mm
Alpaslan 2022 [32]	≥ 4 mm	Yes	≥ 2 mm
Alqahtani 2019 [36]	≥ 4 mm	-	≥ 3 mm
Alqahtani 2020 [37]	≥ 4 mm	-	≥ 3 mm
Arisan 2015 [29]	≥ 4 mm	Yes	≥ 3 mm
Laleman 2020 [25]	≥ 4 mm	Yes	≥ 1 mm
Roos-Jansåker 2017 [38]	≥ 4 mm	Yes	≥ 2 mm
Machtei 2021 [26]	≥ 5 mm	Yes	≥ 3 mm
Merli 2020 [31]	≥ 5 mm	Yes	≥ 1 mm
Park 2021 [39]	≥ 5 mm	Yes	≥ 1 mm
Polymeri 2022 [33]	≥ 5 mm	Yes	≥ 3 mm
Roccuzzo 2022 [34]	≥ 5 mm	Yes	≥ 2 mm
Shibli 2019 [28]	≥ 5 mm	Yes	≥ 4 mm
Strauss 2021 [30]	≥ 5 mm	Yes	≥ 1 mm
Blanco 2022 [17]	≥ 6 mm	Yes	≥ 3 mm
Machtei 2012 [27]	≥ 6 mm	Yes	≥ 1 mm

The studies were grouped according to the PPD defined as a cut-off for the diagnosis. *PPD* Pocket Probing Depth, *BoP* Bleeding on Probing, *MBL* Marginal Bone Loss

### Risk of bias in studies

**Fig. 2:**
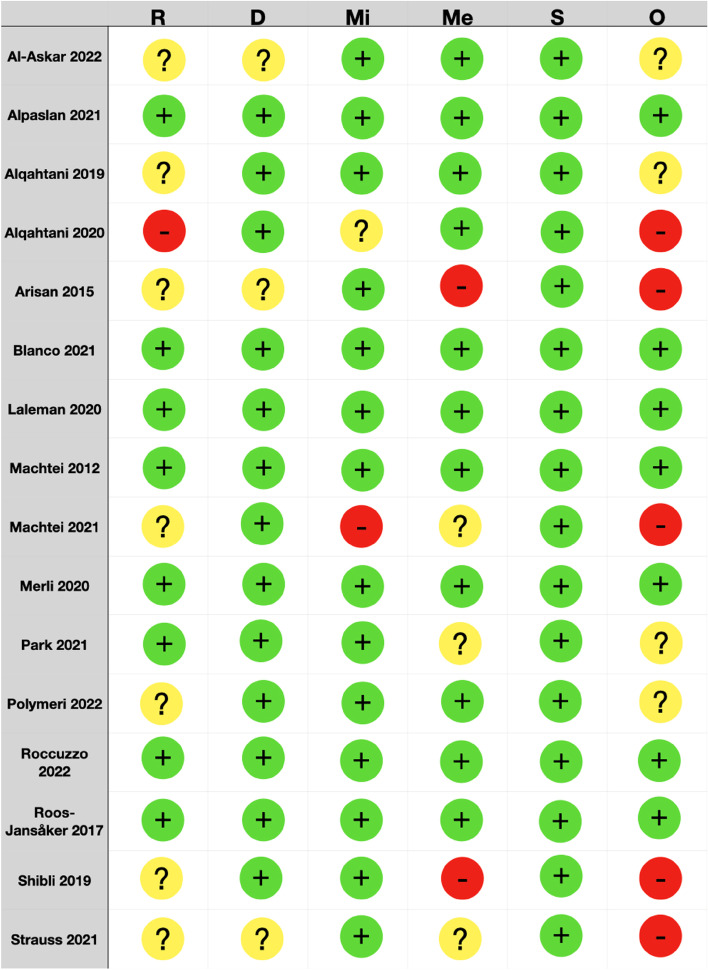
Risk of Bias of the included studies (RoB 2 tool according to Sterne et al.). Risk of bias legend: R: bias arising from the randomization process; D: bias due to deviations from intended interventions; Mi: bias due to missing outcome data; Me: bias in measurement of the outcome; S: bias in selection of the reported results; O: overall risk of bias. Judgements:  Low risk of bias;  High risk of bias;  Some Concerns

Out of 16 included studies, 5 were rated at high risk of bias, 4 at unclear risk of bias, and 7 at low risk of bias (Fig. 2).

### Results of syntheses

**Fig. 3:**
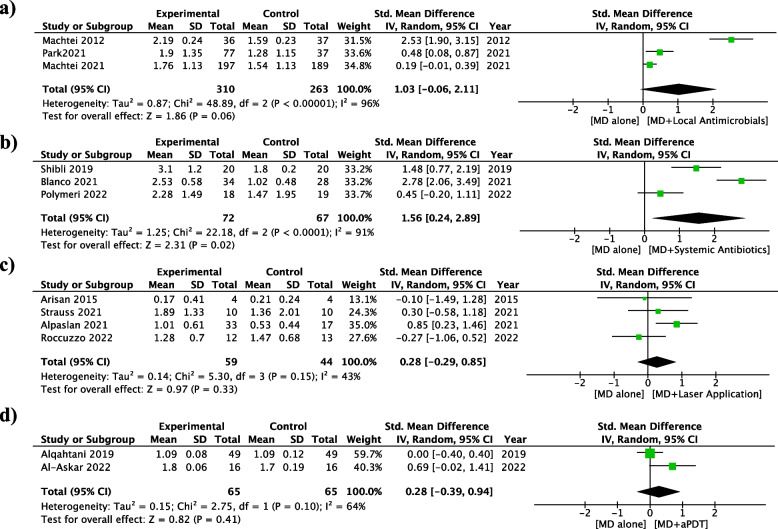
Meta-analyses of the included studies: mean difference in PPD Reduction. MD: Debridement of the implant surface. **a** MD alone vs MD + Local Antimicrobials. **b** MD alone vs MD + Systemic Antibiotics. **c** MD alone vs MD + Laser Application. **d** MD alone vs MD + aPDT

**Fig. 4:**
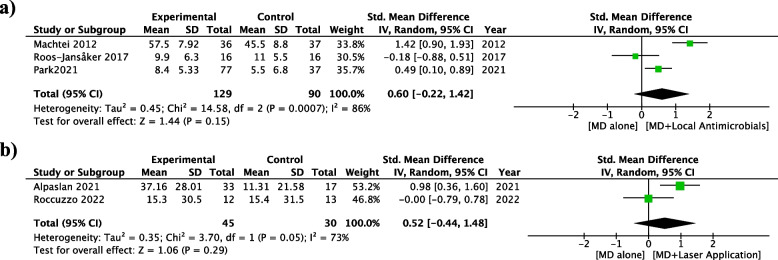
Meta-analyses of the included studies: mean difference in BoP Reduction. MD: Debridement of the implant surface **a** MD alone vs MD + Local Antimicrobials; **b** MD alone vs MD + Laser Application

**Fig. 5:**
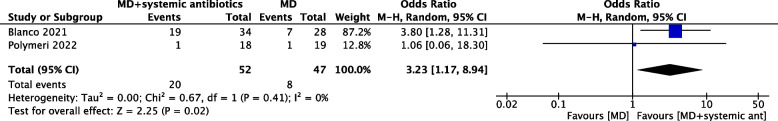
Meta-analysis of the included studies: OR for Resolution of peri-implantitis. MD: Debridement of the implant surface

Thirteen RCTs were included in the meta-analyses and a total of seven meta-analyses were performed (Figs. 3, 4 and 5).

The use of systemic antimicrobials was the only adjunctive method which provided a statistically significant PPD reduction (1.56 mm; [95% CI 0.24 to 2.89]; *p* = 0.02), with higher probability of treatment success (OR = 3.23; [95% CI 1.17 to 8.94]; *p* = 0.02), compared to debridement alone. The adjunctive use of local antimicrobials did not provide additional benefit for PPD reduction (1.03 mm; [95% CI -0.06 to 2.11]; *p* = 0.06) and BoP reduction (0.6%; [95% CI -0.22 to 1.42]; *p* = 0.43) compared to debridement alone. Furthermore, the use of Lasers was not associated to PPD (0.28 mm; [95% CI -0.29 to 0.85]; *p* = 0.33) or BoP reduction (0.52%; [95% CI -0.44 to 1.48]; *p* = 0.29) and aPDT for PPD reduction (0.28 mm; [95% CI -0.39 to 0.94]; *p* = 0.41).

The heterogeneity was considerable in 3 meta-analyses: PPD Reduction for Mechanical Debridement (MD) vs MD + local antimicrobials (I^2^ = 96%; *p* < 0.05), PPD reduction for MD vs MD + systemic antibiotics (91%; *p* < 0.05) and BoP Reduction for MD vs local antimicrobials (86%; *p* < 0.05).

## Discussion

### Summary of main findings

The findings of the present SR showed that only 2 implants out of 1089 were lost at the final follow-up (3–12 months). However, this should be considered as potential proof of success with caution. In fact, peri-implant health is defined as the absence of inflammation, absence of BoP and no evidence of increased PPD/bone loss following initial healing [40]. When a composite outcome (i.e., minimal PPD, no BoP, no progressive bone loss) was considered to define treatment success after non-surgical treatment, the rate of resolution was low and unpredictable (from 5.3 to 57.1%). Additionally, follow-up was short (3 to 12 months), and no data were available in the long-term. This could be explained by the low-resolution rate after non-surgical treatment and the subsequent need for surgery, which made very difficult to have medium and long-term data on implant survival using non-surgical approaches. Thus, non-surgical treatment associated or not to adjunctive methods cannot be considered predictable for the resolution of peri-implantitis in the long-term, avoiding implant loss.

Nevertheless, non-surgical treatment was associated to reduction of peri-implant pocket depth. All the studies included in this SR reported PPD reduction after implant debridement (alone) that varied from 0.2 mm to 1.8 mm. This heterogeneity in the outcomes may be explained by some elements including different conditions at baseline and diverse ability of operators. Even if data are limited, pocket-related response may be also expected, since higher PPD reduction at 3 months for ≥ 7 mm pockets (2.78 mm) compared to 4-6 mm pockets (1.24 mm) was described in a trial [2]. Baseline mean PPD values in this SR were heterogeneous (4.14 mm to 8 mm), even though there were no significant differences between groups in the single studies.

Both peri-implant mucositis and peri-implantitis are characterized by tissues inflammation. Even though it is difficult to define the role of the tissue inflammation on the progression from mucositis to peri-implantitis, the evolution of patients affected by peri-implant mucositis was evaluated in a retrospective study [6]. After 5 years, BoP at more than 50% of the sites and PPD ≥ 4 mm at more than 5% of the sites were associated to risk of progression to peri-implantitis. It could be speculated that prolonged tissue inflammation is a main risk factor for progression. In this SR, non-surgical treatment was effective in reducing BoP values between 5.3 to 57.1%. However, results were heterogeneous and residual inflammation was present at majority of treated implants. Thus, non-surgical therapy should be considered unpredictable in reducing BoP at peri-implantitis site.

### Agreements and disagreements with other SR and studies

A previous SR with a Bayesian network meta-analysis was performed by Faggion et al. in 2014, including 11 studies [41]. The aim was to compare the clinical effect of various non-surgical peri-implantitis therapies. The authors concluded that the evidence was not sufficient to support the superiority of any treatment. Nevertheless, MD + antibiotics achieved an estimate difference of 0.490 mm for PPD reduction in comparison to debridement alone. Despite the differences in methodology, our results are in agreement with their SR.

Systemic antibiotics determined a significant PPD reduction with a difference of 1.56 mm (*p* < 0.02) compared to debridement alone in this SR. Similarly, a difference of 1.46 mm favoring adjunctive systemic antibiotics was reported in another SR [42]. Contrary, different results were found in a recent RCT by De Waal et al. [2]. In their RCT, peri-implantitis was treated by means of full-mouth mechanical debridement and air-powder (erythritol powder containing chlorhexidine) in the control group, while test group received adjunctive systemic AMX/MTZ also. After 3 months, clinical conditions improved in both groups, but no significant differences for any outcome were reported. It could be speculated that other adjunctive methods or combinations may reach similar PPD reduction compared to systemic antibiotics.

Nevertheless, it is mandatory to analyze the clinical effectiveness in terms of defect resolution and further need of additional surgery. In our SR, the use of systemic antibiotics determined a threefold increase of treatment success chance.

This result should be interpreted with extreme caution because, looking into the data retrieved from the single studies, the number of diseased implants after non-surgical therapy remained high and there was a consistent heterogeneity among the proposed experimental treatments. Additionally, no data were available in the long-term, since included RTCs had a maximum 1-year follow-up. Within this context, it is very difficult to understand the role of adjunctive systemic antibiotics in reducing the need for surgery. Moreover, it is worth to mention the issue of antibiotic resistance. The subgingival peri-implant pathogens were found to be resistant in vitro to individual concentration of clindamycin, amoxicillin, doxycycline, or metronidazole in 71.7% of the subjects [43]. Finally, all the studies used metronidazole, and its administration for oral infection is not allowed in all countries.

The local application of antimicrobials was associated to a not statistically significant difference in terms of PPD reduction (1.03 mm; [95% CI -0.06 to 2.11]; *p* = 0.06) compared to debridement alone. The heterogeneity was considerable. Two of the studies included in this meta-analysis were multicenter RCTs and tested repeated applications of chlorhexidine chips. Another trial tested the efficacy of minocycline and/or metronidazole ointments. Our results are similar to those reported in a previous SR on peri-implantitis [41] and in a more recent SR focusing on peri-implant mucositis [44]. Thus, the efficacy of repeated applications of chlorhexidine chips or minocycline and/or metronidazole remains controversial.

This SR failed to demonstrate significant clinical benefit for adjunctive use of lasers for both PPD (0.28 mm; [95% CI -0.29 to 0.85]; *p* = 0.33) and BoP reduction (0.52%; [95% CI -0.44 to 1.48]; *p* = 0.29). In accordance with these results, a recent SR found only minimal benefit using lasers in terms of PPD reduction (0.15 mm) after non-surgical therapy [45]. Similarly, a previous SR [46], aiming to evaluate the effectiveness of laser therapy in managing peri-implant mucositis and peri-implantitis, failed to reveal any superiority when laser treatment was performed, alone or as an adjunctive.

A RCT found higher BoP reduction with the adjunctive use of a Er:YAG laser after 6 months, however after 12 months no significant differences were reported [47]. It could be hypothesized that self-performed oral hygiene and adherence to supportive peri-implant therapy may be more important for controlling the bacterial colonization of the peri-implant pocket that the treatment itself.

Although in vitro studies showed that aPDT may be effective in bacterial killing on titanium surfaces [48], clinical improvement in terms of PPD reduction was minimal for aPDT (0.33 mm; [95% CI -0.34 to 1.01]; *p* = 0.33) in this SR, and this data are in accordance with a recently published SR [49].

It could be hypothesized that, even if aPDT may be effective in bacterial killing having an initial effect, bacterial recolonization of the peri-implant pocket is not prevented especially in case on incomplete resolution [50]. In fact, peri-implantitis resolution was under 50% for adjunctive PDT, lasers or chlorhexidine (CHX) [31, 51, 52]. Contrary, another SR [42] found that adjunctive aPDT therapy led to significant PD reduction over a 6-month period compared to the mechanical debridement alone. However, this conclusion was based upon the analysis of a single study [53] that was not included in our SR. Therefore, the clinical efficacy of adjunctive aPDT remains controversial.

### Quality of the evidence and potential limitations in the review process

This SR has several limitations. It included only studies in which the control group was the debridement alone, in order to reduce the potential source of bias and have a clear view of the benefits of adjunctive methods; thus, direct comparisons between different adjunctive therapies were not possible. Furthermore, at the time the included RCTs were performed, there were huge differences in defining peri-implantitis. Different criteria for the diagnosis of peri-implantitis also implies differences in baseline PPD that may have influenced the results of pooled estimates. Unfortunately, subgroup analyses stratified according to baseline PPD were not considered appropriate, due to the paucity of studies included in each meta-analysis.

Finally, even though composite outcomes could be considered appropriate to define the efficacy of the therapy, only short-follow-up data were available and very few studies used composite outcomes (i.e., PPD and BoP and MBL) to report disease resolution. Future RCTs should consider composite outcomes to define disease resolution, in order to improve the understanding of the clinical effectiveness of peri-implantitis methods and therapies.

Another factor that may jeopardize the results of our SR lies in the risk of bias in the included studies. Although every effort was made to include high quality papers, only 7 RCTs were rated at low risk of bias. Future studies should provide a better description of the randomization process and of the allocation concealment, which were frequently omitted.

Finally, another limitation of this SR lies in the fact that half of the included papers did not provide any information about the implant surfaces. Machined implants were treated in one study [28], while six studies treated “moderate rough” surfaces without specifying the implant brand [17, 27, 35–37] except for the study from Roccuzzo [34]. The biofilm removal may be difficult due to geometry of the threads, and the presence of irregular rough/porous titanium surface made difficult to obtain the complete elimination of the bacterial biofilm, jeopardizing the clinical results. Future studies should better describe these data, in order to understand how much the implant characteristics influence the clinical results.

## Conclusions

Withing the limits of this SR, it can be concluded that:

- Non-surgical treatment of peri-implantitis, with or without the use adjunctive methods, may reduce PPD and BoP
- Complete disease resolution is unpredictable to achieve with non-surgical peri-implant therapy.
- The adjunctive use of systemic antibiotics seems to improve the efficacy of MD, but their use should be considered with caution in routine practice.
- Minimal benefit was found for the adjunctive use of laser and aPDT, when compared to MD alone.

## Implications for practice and future research

The clinical benefit of adjunctive lasers, aPDT and local antimicrobial therapies for NST of peri-implantitis remains unclear and should not be used routinely. Systemic AMX/MTZ may improve PPD reduction and could be considered in specific cases.

Future studies should be designed considering peri-implant disease resolution/health defined as composite outcome (PPD, BOP, MBL) to better understand the role of adjunctive therapy.

## Supplementary Information

**Additional file 1.**

**Additional file 2.**

**Additional file 3.**

**Additional file 4.**

## Data Availability

The authors confirm that the data supporting the findings of this study are available within the article and its supplementary materials.

## Abbreviations

BoP: Bleeding on Probing
PPD: Pocket Probing Depth
aPDT: Antimicrobial PhotoDynamic Therapy
SR: Systematic Review
NST: Non-Surgical Treatment
RAL: Relative clinical Attachment Level
MBL: Marginal Bone Level
RCT: Randomized Clinical Trial
WMD: Weighted Mean Differences
CI: Confidence Interval
OR: Odds Ratio
MD: Mechanical Debridement
CHX: Chlorhexidine
