# Supplementary material for: Clinical efficacy of adjunctive methods for the non-surgical treatment of peri-implantitis: a systematic review and meta-analysis
Source: BMC Oral Health. 2023 Jun 9;23:375. doi: 10.1186/s12903-023-03058-z (PMC10251565; doi:10.1186/s12903-023-03058-z)
Supplement: Supplementary file 1 — Additional file 1. [file 12903_2023_3058_MOESM1_ESM.docx]

**Additional file 1**

**Search strategy in *MEDLINE* (via PubMed) on October 20, 2022**

1. “Peri-implantitis"[Mesh] [Tw] OR "Peri-implant disease" [Tw] OR "peri-implant mucositis" [Tw] AND
2. "Non-surgical therapy" [Tw] OR "non-surgical treatment" [Tw] OR "anti-infective treatment" [Tw] OR "curettage" [Tw] OR "non-surgical debridement" [Tw] OR "non-surgical mechanical debridement therapy" [Tw] OR "non-surgical intervention" [Tw] OR "scaling and root planning" [Tw] OR "SRP" [Tw] OR "full mouth debridement" [Tw] OR "Debridement"[Tw] AND
3. "Local anti-infective agents" [Tw] OR "laser therapy" [Tw] OR "photo-chemotherapy" [Tw] OR "antibiotics" [Tw]

**LIMITS**:

- - Type of Article: Randomized Controlled Trial.
  - Dates: until 20.10.2022.
  - Humans or Animals: Humans.

**Search strategy in EMBASE on October 20, 2022**

1. “Peri-implantitis"[Mesh] OR "Peri-implantitis" [Tw] OR "Peri-implant disease" [Tw] OR "peri-implant mucositis" [Tw] AND
2. "Non-surgical therapy" [Tw] OR "non-surgical treatment" [Tw] OR "anti-infective treatment" [Tw] OR "curettage" [Tw] OR "non-surgical debridement" [Tw] OR "non-surgical mechanical debridement therapy" [Tw] OR "non-surgical intervention" [Tw] OR "scaling and root planning" [Tw] OR "SRP" [Tw] OR "full mouth debridement" [Tw] OR "Debridement"[Tw] AND
3. "Local anti-infective agents" [Tw] OR "laser therapy" [Tw] OR "photo-chemotherapy" [Tw] OR "antibiotics" [Tw]

**LIMITS**:

- 1. Type of Article: Randomized Controlled Trial.
  2. Dates: until 20.10.2022.
  3. Humans or Animals: Humans.

**Search strategy in Cochrane on October 20, 2022**

1. “Peri-implantitis"[Mesh] OR "Peri-implantitis" [Tw] OR "Peri-implant disease" [Tw] OR "peri-implant mucositis" [Tw] AND
2. "Non-surgical therapy" [Tw] OR "non-surgical treatment" [Tw] OR "anti-infective treatment" [Tw] OR "curettage" [Tw] OR "non-surgical debridement" [Tw] OR "non-surgical mechanical debridement therapy" [Tw] OR "non-surgical intervention" [Tw] OR "scaling and root planning" [Tw] OR "SRP" [Tw] OR "full mouth debridement" [Tw] OR "Debridement"[Tw] AND
3. "Local anti-infective agents" [Tw] OR "laser therapy" [Tw] OR "photo-chemotherapy" [Tw] OR "antibiotics" [Tw]
   1. Dates: until 20.10.2022.
   2. Humans or Animals: Humans.

**Legend**. **MESH**: MEdical Subject Heading; **Tw**: Text word.
