# Supplementary material for: Clinical efficacy of adjunctive methods for the non-surgical treatment of peri-implantitis: a systematic review and meta-analysis
Source: BMC Oral Health. 2023 Jun 9;23:375. doi: 10.1186/s12903-023-03058-z (PMC10251565; doi:10.1186/s12903-023-03058-z)
Supplement: Supplementary file 2 — Additional file 2. [file 12903_2023_3058_MOESM2_ESM.docx]

**Additional file 2.** Excluded full-text articles (with reason for exclusion).

| **Reason for exclusion** | **Author (year)** |
| --- | --- |
| Not RCTs | Al Amri et al. 2020, Heo et al. 2018, Lerario et al. 2016, Mettraux et al. 2016, Mombelli et al. 2001, Salvi et al. 2007, Schwarz et al. 2006, Levin et al. 2015 |
| Study on peri-implant mucositis/Unclear definition of peri-implantitis | Abduljiabbar et al. 2017, Al Ghazal et al. 2017, Gomi et al. 2015, Javed et al. 2016, Kashefimehr et al. 2017, McKenna et al. 2013 |
| Do not meet the inclusion criteria for PICO (interventions and comparisons) | Bassetti et al. 2014, De Waal et al. 2021, Esposito et al. 2013, Hentenaar et al. 2021, John et al. 2015, Karring et al. 2005, Mussano et al. 2013, Odatsu et al. 2020, Persson et al. 2011, Pranno et al. 2021, Renvert et al. 2006, Renvert et al. 2008, Renvert et al. 2009, Renvert et al. 2011, Sahm et al 2011, Schär et al. 2013, Schwarz et al. 2005, Schwarz et al. 2006, Tada et al. 2018, Tian et al. 2019, Wagner et al. 2021, Wang et al. 2019, Xu et al. 2016 |
| All the included patients were affected by diabetes | Labban et al. 2021 |
| Short Follow-up | Wei et al. 2020 |
