# Supplementary material for: Clinical efficacy of adjunctive methods for the non-surgical treatment of peri-implantitis: a systematic review and meta-analysis
Source: BMC Oral Health. 2023 Jun 9;23:375. doi: 10.1186/s12903-023-03058-z (PMC10251565; doi:10.1186/s12903-023-03058-z)
Supplement: Supplementary file 3 — Additional file 3. [file 12903_2023_3058_MOESM3_ESM.docx]

**Additional file 3.** Additional information for the included studies

| **Authors** | **Country** | **Setting (Private/Public)** | **Funding** |
| --- | --- | --- | --- |
| **Al-Askar 2022** | Saudi Arabia | University Hospital | No funding was received for this article |
| **Alpaslan 2021** | Turkey | University Hospital | This study was funded by the authors and by Van Yuzuncu Yil University, Van, Turkey |
| **Arisan 2015** | Turkey | University Hospital | This study was supported by a grant from Istanbul University Research Fund |
| **Alqahtani 2019** | Saudi Arabia | University Hospital | The authors are grateful to the Deanship of Scientific Research at King Saud University, Riyadh, Saudi Arabia for funding this research through Vice Deanship of Scientific Research Chairs |
| **Alqhatani 2020** | Saudi Arabia | University Hospital | The authors are grateful to the deanship of Scientific Research,King Saud University, for funding through Vice Deanship of Scientific Research Chairs. |
| **Blanco 2021** | Spain | University Hospital | This study was partially supported by grants from Osteology Foundation (16-070) and from the Spanish Society of Periodontology SEPA |
| **Laleman 2020** | Belgium | University Hospital | This study was partially financially supported by BioGaia AB Sweden. Additional support came from grants from the KU Leuven (C24/17/086) and the FWO (G091218N) |
| **Machtei 2012** | Israel | University Hospital | This study was supported by a research grant from Dexcel Pharma |
| **Machtei 2021** | Israel | University Hospital | This study was supported by a research grant from Dexcel Pharma |
| **Merli 2020** | Italy | Private and Public | - |
| **Park 2021** | South Korea | University Hospital | The study was financially supported by Yuhan Inc. |
| **Polymeri 2022** | Netherlands | University Hospital | This study was supported by the Department of Periodontology and the Department of Oral Implantology and Prosthetic Dentistry, Academic Centre for Dentistry Amsterdam (ACTA) |
| **Roccuzzo 2022** | Switzerland | University Hospital | The study was supported by a small grant of the International Team of Implantology (ITI) |
| **Roos-Jansåker 2017** | Sweden | University Hospital | This study was supported funded by RLS Global AB,  Gothenburg, Sweden. |
| **Shibli 2019** | Brazil | University Hospital | This study was supported by Grant FAPESP (São Paulo Research Foundation) |
| **Strauss 2021** | USA | Private and Public | - |
