# Supplementary material for: Clinical efficacy of adjunctive methods for the non-surgical treatment of peri-implantitis: a systematic review and meta-analysis
Source: BMC Oral Health. 2023 Jun 9;23:375. doi: 10.1186/s12903-023-03058-z (PMC10251565; doi:10.1186/s12903-023-03058-z)
Supplement: Supplementary file 4 — Additional file 4. [file 12903_2023_3058_MOESM4_ESM.docx]

**Additional file 4**. Results of the individual studies. **F.up***:* follow-up; **PPD**: pocket probing depth; **BoP**: Bleeding on Probing; **RAL**: Relative Attachment Level; **MBL**: Marginal Bone Loss; **MD**: Mechanical Debridement; **CHXc**: Chlorhexidine chips; **SA**: Systemic Antibiotics; **MTZ**: Metronidazole; **dLaser**: diode Laser; **Nd:YAG**: neodymium-doped yttrium aluminum garnet; **Dm**: Dessiccant Material; **Gp**: Glycine Powder; **Er,Cr:YSGG**: Erbium, chromium-doped yttrium, scandium, gallium garnet; **AMX**: Amoxicilline; **PBMT**: Photobiomodulation Therapy; **PDT**: Photodynamic Therapy; **aPDT**: antimicrobial photodynamic therapy; **MIN**: Minocycline.

| **Authors** | **N° of patient**  **(n° of implants)** | | **F.up**  **(months)** | **Treatment** | | **Initial PPD**  **(mm, SD)** | | **Initial BoP**  **(%, SD)** | | **RAL gain**  **(mm, SD)** | | **MBL changes**  **(mm, SD)** | |
| --- | --- | --- | --- | --- | --- | --- | --- | --- | --- | --- | --- | --- | --- |
|  | Control | Test |  | Control | Test | Control | Test | Control | Test | Control | Test | Control | Test |
| Al – Askar 2022 | 16 (16) | 17 (17) | 3 | MD | MD + PBMT | 4.4 (0.2) | 4.5 (0.3) | - | - | - | - | - | - |
|  |  | 16 (16) | 3 | MD | MD + PDT |  | 4.2 (0.1) | - | - | - | - | - | - |
| Alpaslan 2021 | 17 (17) | 33 (33) | 6 | MD | MD + Laser (Er,Cr:YSGG/dLaser) | 4.14 (0.64) | 4.31 (0.97) | 72 (23.93) | 94.1 (12.8) | - | - | - | - |
| Alqahtani 2019 | 49 (49) | 49 (49) | 6 | MD | MD + aPDT | 4.84 (0.3) | 4.84 (0.3) | 23.4 (3.5) | 23.4 (3.5) | - | - | 0.1 (0.3) | 0.2 (0.2) |
| Alqahtani 2020 ^a^ | 34 (34) | 33 (33) | 6 | MD | MD + PBMT | - | - | - | - | - | - | - | - |
| Arisan 2015 | 5 (24) | 5 (24) | 6 | MD | MD + dLaser | 5.37 (0.49) | 4.71 (0.67) | 100 | 100 | - | . | 0.28 | 0.66 |
| Blanco 2021 | 16 (28) | 16 (34) | 12 | MD | MD + SA (MTZ) | 6.32 | 6.78 | - | - | 0.53 | 2.14 | 1.13 | 2.33 |
| Laleman 2020 | 8 (8) | 6 (6) | 6 | MD | MD + Probiotic drop | 5.45 (1.20) | 5.17 (0.92) | 87 (22) | 87 (23%) | - | - | - | - |
| Machtei 2012 | 30 (37) | 26 (36) | 6 | MD | MD + CHXc | 7.21 | 7.60 | 100 | 100 | 1.56 (0.25) | 2.21 (0.23) | - | - |
| Machtei 2021 | 144 (189) | 146 (197) | 6 | MD | MD + CHXc | 6.06 (0.92) | 6.16 (1.00) | 100 | 100 | 1.39 (1.27) | 1.47 (1.32) | - | - |
| Merli 2020 | 16 (15) | 15 (15) | 6 | MD | MD + Dm | 4.4 (1.1) | 5(1.2) | 55 (13.3) | 48.3 (21.7) | 0.1 (0.6) | 0.6 (0.9) | - | - |
|  |  | 13 (15) | 6 |  | MD + Gp | - | 5.1 (1.5) | - | 60 (13.3) | - | 0.1 (0.9) | - | - |
|  |  | 14 (15) | 6 |  | MD + Gp + Dm | - | 4.9 (1.1) | - | 60 (13.3) | - | 0.7 (0.8) | - | - |
| Park 2021 | 37 (37) | 38 (38) | 3 | MD | MD + MTZ + MIN | 5.71 (1.33) | 5.71 (1.33) | 13.3 (4.2) | 13.8 (3.6) | - | - | - | - |
|  |  | 39 (39) | 3 |  | MD + MIN |  | 6.22 (1.92) |  | 13.6 (4) | - | - | - | - |
| Polymeri 2022 | 19 (19) | 18 (18) | 3 | MD | MD + SA (AMX+MTZ) | 8 (1.41) | 7.44 (1.38) | - | - | - | - | - | - |
| Roccuzzo 2022 | 13 (13) | 12 (12) | 6 | MD | MD + dLaser | 5.29 (0.52) | 5.40 (0.81) | 62.8 (21.7) | 62.5 (30.3) | - | - | 0.03 (0.23) | 0.04 (0.50) |
| Roos-Jansåker 2017 | 16^*^ (16) | 16 (16) | 3 | MD | MD + Chloramine | 5.25^§^ | 5.38^§^ | 16.7 (2) | 16.7 (2) | 1.9^§^ | 2^§^ | - | - |
| Shibli 2019 | 20 (20) | 20 (20) | 12 | MD | MD + SA (AMX+MTZ) | 5.5 (1.3) | 7.0 (2.6) | 85.0 (18.3) | 86.6 (32.2) | 1.4 (0.8) | 2.6 (1.5) | - | - |
| Strauss 2021 | 10 (15) | 10 (19) | 12 | MD | MD + Laser (Nd:YAG) | 5.34 (2.01) | 6.51 (1.33) | 80 | 100 | - | . | 0.26 (0.64) | 0.41 (0.92) |

^a^ For this study, baseline and final measurements were reported on graphs.

^*^ Split-mouth study. Sixteen patients were the total sample.
